# Supplementary material for: The interaction of DNA repair factors ASCC2 and ASCC3 is affected by somatic cancer mutations
Source: Nat Commun. 2020 Nov 2;11:5535. doi: 10.1038/s41467-020-19221-x (PMC7608686; doi:10.1038/s41467-020-19221-x)
Supplement: Supplementary file 1 — Supplementary Information [file 41467_2020_19221_MOESM1_ESM.pdf]

Supplementary Information

The interaction of DNA repair factors ASCC2 and ASCC3 is affected by somatic cancer mutations

Junqiao Jia[1], Eva Absmeier[1,5], Nicole Holton[1], Agnieszka J. Pietrzyk-Brzezinska[1,6], Philipp Hackert[2], Katherine E. Bohnsack[2], Markus T. Bohnsack[2,3], Markus C. Wahl[1,4,*]

[1] Freie Universität Berlin, Laboratory of Structural Biochemistry, Takustraβe 6, D-14195 Berlin, Germany

[2] University Medical Centre Göttingen, Department of Molecular Biology, Göttingen, Germany

[3] Georg-August-Universität, Göttingen Center for Molecular Biosciences, Göttingen, Germany

[4] Helmholtz-Zentrum Berlin für Materialien und Energie, Macromolecular Crystallography, Albert-Einstein-Straße 15, D-12489 Berlin, Germany

[5] Present address: MRC Laboratory of Molecular Biology, Cambridge Biomedical Campus, Francis Crick Avenue, Cambridge, CB2 0QH, United Kingdom

[6] Present address: Institute of Molecular and Industrial Biotechnology, Faculty of Biotechnology and Food Sciences, Lodz University of Technology, Lodz, 90-924, Poland

* Correspondence to: markus.wahl@fu-berlin.de

Supplementary Tables

Supplementary Table 1: Crystallographic data.

**Data collection**	
Wavelength [Å]	0.97957
Space group	I4
Unit cell parameters	
a (= b), c [Å]	158.1, 69.4
Resolution [Å][a]	50.0-2.7 (2.86-2.70)
Reflections	
Total	162,207 (26,160)
Unique	45,845 (7,271)
Multiplicity	3.5 (3.6)
Completeness [%]	99.0 (97.8)
Mean I / $\sigma(I)$	5.89 (0.47)
$R_{merge}(I)$ [%][(b)]	16.4 (243.5)
$R_{meas}(I)$ [%][(c)]	19.3 (285.9)
$CC_{1/2}$ [%][(d)]	99.5 (13.9)
**Phasing**	
Resolution [Å]	42.0-2.8
Number of selenium atoms	6
Figure of merit	0.2
BAYES-CC	22.9
**Refinement**	
Resolution [Å][a]	42.0-2.7 (2.76-2.70)
Reflections	
Unique	23,723 (2,342)
Test set [%]	4.6 (4.6)
R_{work} [%][(e)]	20.4 (33.5)
R_{free} [%][(f)]	24.7 (34.8)
Contents of A.U.[(g)]	
Non-H atoms	4,784
Protein residues/atoms	584/4,729
Water oxygens	55
Mean B factors [Å^2]	
Wilson	86.1
Model atoms	96.0
Rmsd[(h)] from ideal geometry	
Bond lengths [Å]	0.007
Bond angles [°]	0.92
Model quality[(i)]	
Overall score	1.9
Clash score	11.0
Ramachandran favored [%]	95.2
Ramachandran outliers [%]	0.7
PDB ID	6YXQ

[a] Values in parentheses refer to the highest resolution shell.

[b] $R_{merge}(I) = \sum_h \sum_i |I_{ih} - \langle I_h \rangle| / \sum_h \sum_i I_{ih}$, in which $\langle I_h \rangle$ is the mean intensity of symmetry-equivalent reflections h and I_{ih} is the intensity of a particular observation of h.[55]

[c] $R_{meas}(I) = \sum_h [N / (N-1)]^{1/2} \sum_i |I_{ih} - \langle I_h \rangle| / \sum_h \sum_i I_{ih}$, in which $\langle I_h \rangle$ is the mean intensity of symmetry-equivalent reflections h, I_{ih} is the intensity of a particular observation of h and N is the number of redundant observations of reflection h.[55]

[d] $CC_{1/2} = (\langle I^2 \rangle - \langle I \rangle^2) / (\langle I^2 \rangle - \langle I \rangle^2) + \sigma^2_\varepsilon$, in which σ^2_ε is the mean error within a half-dataset.[55]

[e] $R_{work} = \sum_h |F_o - F_c| / \sum F_o$ (working set, no σ cut-off applied).

[f] R_{free} is the same as R_{work}, but calculated on the test set of reflections excluded from refinement.

[g] A.U. – asymmetric unit.

[h] Rmsd – root-mean-square deviation

[i] Calculated with MolProbity[56].

Supplementary Table 2: PCR primers.

Primer name[a]	Sequence
Slic-sumo-F	CCACCATCGGGCGCGGATCCATGCACCATCACCATCACCA
Slic-sumo-R	ATGTCGTAATCGCTCATGGGGCCGCCGGTTTGTTCCTGGT
Slic-ASCC3^{FL}-F	ACCAGGAACAAACCGGCGGCCCCATGAGCGATTACGACAT
Slic-ASCC3^{FL}-R	TGGTGATGGTGATGGTGCATGGATCCGCGCCCGATGGTGG
pFL-ASCC3^{NTR}-F	GGTTTTGAATTCATGGCACTTCCAAGGCTTAC
pFL-ASCC3^{NTR}-R	GGTTTTAAGCTTTTAGATCTTCTCTACATCAGCATCC
pFL-ASCC3^{HR}-F	TCTTTATTTTCAGGGCGCCGAATTCCACTACCCTCACGTTTACGACTCAC
pFL-ASCC3^{HR}-R	TCCTCTAGTACTTCTCGACAAGCTTTTATTTCAGGGCGAGGTCGGT
pFL-ASCC3^{NC}-F	TCTTTATTTTCAGGGCGCCGAATTCCACTACCCTCACGTTTACGACTCAC
pFL-ASCC3^{NC}-R	TCCTCTAGTACTTCTCGACAAGCTTTTATGTATGTGGTGGATGCCTCTCTG
pFL-ASCC3$^{1-207}$-F	GGTTTTGAATTCATGGCACTTCCAAGGCTTAC
pFL-ASCC3$^{1-207}$-R	GGTTTTAAGCTTTTA TGCCTCTTGCAGGTGCTCA
pFL-ASCC3$^{1-197}$-F	GGTTTTGAATTCATGGCACTTCCAAGGCTTAC
pFL-ASCC3$^{1-197}$-R	GGTTTTAAGCTTTTACTTGTAGTCGAGGCTGATGGT
pFL-ASCC3$^{1-160}$-F	GGTTTTGAATTCATGGCACTTCCAAGGCTTAC
pFL-ASCC3$^{1-160}$-R	GGTTTTAAGCTTTTATCTGTCACCGTGCTCCTTC
pFL-ASCC2^{FL}-F	TCTTTATTTTCAGGGCGCCGAATTCATGCCAGCTCTTCCACTGGAT
pFL-ASCC2^{FL}-R	TCCTCTAGTACTTCTCGACAAGCTTTTAAGAGGGAATCATGCCTTTCGAG
pUCDM-ASCC2$^{1-434}$-F	GGTTTTGGATCCTATGCCAGCTCTTCCACTGG
pUCDM-ASCC2$^{1-434}$-R	GGTTTTTCTAGATTAGGCTGTCACGGTTACACCG
pFL-ASCC3$^{HR-K505N}$-F	CCCCTACGGGAGCTGGAAATACTAACATCGCTATG
pFL-ASCC3$^{HR-K505N}$-R	CATAGCGATGTTAGTATTTCCAGCTCCCGTAGGGG
pFL-ASCC3$^{HR-K1355N}$-F	GTGCTCCAACAGGTAGTGGAAATACAGTAGCTGCT
pFL-ASCC3$^{HR-K1355N}$-R	AGCAGCTACTGTATTTCCACTACCTGTTGGAGCAC
pFL-ASCC3$^{HR-D611A}$-F	CAGGCTGCTGATTCTCGCTGAAGTACACCTCTTGC
pFL-ASCC3$^{HR-D611A}$-R	GCAAGAGGTGTACTTCAGCGAGAATCAGCAGCCTG
pFL-ASCC3$^{HR-D1453A}$-F	AACGATCCTGATCATTGCCGAGATCCACCTGTTGG
pFL-ASCC3$^{HR-D1453A}$-R	CCAACAGGTGGATCTCGGCAATGATCAGGATCGTT
pETM11-ASCC3^{NTR}-F	GGTTTTCCATGGCACTTCCAAGGCTTAC
pETM11-ASCC3^{NTR}-R	GGTTTTAAGCTTTTAGATCTTCTCTACATCAGCATCC
pETM11-ASCC3$^{1-161}$-F	GGTTTTCCATGGCACTTCCAAGGCTTAC
pETM11-ASCC3$^{1-161}$-R	GGTTTAAGCTTTTAAACTCTGTCACCGTGCTC
pETM11-ASCC3$^{16-197}$-F	GGTTTCCATGGCGGTAACGAAGCAGGATAACTACA
pETM11-ASCC3$^{16-197}$-R	GGTTTTAAGCTTTTACTTGTAGTCGAGGCTGATGGT
pETM11-ASCC3$^{42-197}$-F	GGTTTCCATGGCGGACCTTGGACTGACATGGAA
pETM11-ASCC3$^{42-197}$-R	GGTTTTAAGCTTTTACTTGTAGTCGAGGCTGATGGT
pETM11-ASCC2$^{1-434}$-F	GGTTTCCATGGCGATGCCAGCTCTTCCACTGG
pETM11-ASCC2$^{1-434}$-R	GGTTTTAAGCTTTTAGGCTGTCACGGTTACACCG

[a] F, forward; R, reverse

Supplementary Table 3: Synthetic genes sequences[a].

Synthetic gene sequence for expression of ASCC3^{FL}
ATGGCTCTGCCTAGACTGACTGGTGCCCTGCGCTCTTTCAGCAACGTCACCAAGCAGGACAACTACAACGA
AGAAGTCGCCGACCTGAAGATCAAGCGCTCTAAGCTGCACGAACAGGTCCTGGACTTGGGACTGACCTGGA
AGAAGATCATCAAGTTCCTCAACGAGAAGCTCGAGAAGTCCAAGATGCAGTCCATCAACGAGGACCTGAAG
GACATCCTGCACGCCGCCAAGCAGATCGTCGGAACTGACAACGGAAGGGAAGCCATCGAATCTGGTGCCG
CCTTCCTGTTCATGACCTTCCACCTCAAGGACTCCGTCGGACACAAAGAAACTAAGGCCATCAAGCAGATGT
TCGGCCCCTTCCCATCTTCCTCTGCTACCGCTGCTTGCAACGCCACCAACCGCATCATCTCCCACTTCTCTC
AGGACGACCTGACCGCTCTGGTCCAGATGACCGAAAAAGAACACGGCGACAGGGTGTTCTTCGGAAAGAA
CCTGGCCTTCTCTTTCGACATGCACGACCTGGACCACTTCGACGAACTGCCCATCAACGGCGAAACCCAAA
AGACCATCTCTCTGGACTACAAGAAGTTCTTGAACGAGCACCTCCAAGAGGCTTGCACCCCTGAACTGAAG
CCCGTCGAAAAGACCAACGGATCTTTCCTGTGGTGCGAGGTCGAAAAGTACCTGAACTCTACCCTGAAAGA
GATGACTGAAGTCCCTCGCGTCGAGGACTTGTGCTGCACCCTGTACGACATGCTGGCCTCTATCAAGTCTG
GCGACGAGCTGCAGGACGAACTGTTCGAACTGCTGGGACCCGAAGGACTGGAACTGATCGAAAAGCTGCT
GCAGAACCGCATTACTATCGTGGACCGCTTCCTGAACTCCTCTAACGACCACAGGTTCCAGGCTCTGCAAGA
CAACTGCAAGAAGATCCTGGGAGAAAACGCCAAGCCTAACTACGGATGCCAAGTCACCATCCAGTCTGAGC
AAGAAAAGCAGCTGATGAAGCAGTACCGCCGCGAAGAAAAGAGGATCGCCAGGCGAGAAAAGAAGGCCGG
CGAAGATCTGGAAGTCTCTGAAGGACTGATGTGCTTCGACCCCAAAGAACTGCGCATCCAGCGCGAACAGG
CTCTCCTGAACGCTCGCTCTGTCCCCATCTTGTCTCGTCAGAGGGACGCCGACGTCGAGAAGATTCACTAC
CCTCACGTCTACGACAGCCAGGCCGAAGCCATGAAGACCTCTGCCTTCATTGCCGGTGCCAAGATGATCCT
GCCTGAGGGAATCCAGAGGGAAAACAACAAGCTGTACGAGGAAGTGCGCATCCCCTACTCTGAACCCATGC
CTCTGTCTTTCGAAGAGAAGCCCGTGTACATCCAGGACTTGGACGAAATCGGACAGCTGGCCTTCAAGGGA
ATGAAGAGGCTGAACCGCATCCAGTCCATCGTGTTCGAAACCGCCTACAACACCAACGAGAACATGCTGATC
TGCGCCCCTACCGGTGCCGGCAAGACCAACATTGCTATGCTGACCGTCCTGCACGAAATCAGGCAGCACTT
CCAGCAGGGTGTCATCAAGAAGAACGAGTTCAAGATCGTCTACGTCGCCCCTATGAAGGCCCTGGCCGCTG
AAATGACCGACTACTTCTCTAGGCGCCTCGAACCTCTGGGAATCATCGTCAAAGAACTCACCGGCGACATGC
AGCTGTCTAAGTCTGAAATCCTGAGGACCCAGATGCTGGTCACCACTCCTGAAAAGTGGGACGTCGTCACC
CGCAAGTCTGTCGGAGATGTCGCCCTGTCTCAGATCGTCAGGCTGCTGATCCTGGACGAAGTCCACCTGTT
GCACGAGGACAGAGGACCCGTCCTGGAATCTATCGTCGCTAGGACCCTGAGGCAGGTCGAATCTACCCAGT
CTATGATCCGCATCCTGGGACTGAGCGCTACCCTGCCAAACTACTTGGACGTCGCCACCTTCCTGCACGTCA
ACCCCTACATCGGACTGTTCTTCTTCGACGGACGCTTCCGTCCTGTGCCTCTGGGACAGACCTTCCTCGGA
ATCAAGTGCGCCAACAAGATGCAACAGCTGAACAACATGGATGAAGTCTGCTACGAGAACGTCCTGAAGCAA
GTCAAGGCCGGACACCAAGTCATGGTGTTCGTCCACGCTAGGAACGCCACCGTCAGGACCGCTATGTCTCT
GATCGAACGCGCCAAGAACTGTGGACACATCCCATTCTTCTTCCCAACTCAAGGACACGACTACGTCCTGGC
CGAAAAGCAGGTCCAGAGGTCTAGGAACAAGCAAGTCCGCGAGCTGTTCCCCGACGGATTCTCTATTCACC
ACGCCGGAATGCTGCGCCAGGACCGTAACCTGGTCGAAAACCTGTTCTCTAACGGACACATCAAGGTTCTC
GTCTGCACCGCCACCTTGGCCTGGGGAGTTAACTTACCTGCTCACGCCGTCATCATCAAGGGAACCCAGAT
CTACGCTGCCAAGCGCGGTTCTTTCGTGGACCTGGGAATCCTCGACGTCATGCAAATCTTCGGACGCGCCG
GAAGGCCCCAGTTCGACAAATTCGGAGAAGGCATCATCATCACCACTCACGACAAGCTGTCTCACTACCTGA
CCTTGCTGACCCAGAGGAACCCTATCGAATCTCAGTTCCTGGAAAGCCTGGCCGACAACCTGAACGCCGAA
ATCGCTTTGGGAACCGTCACCAACGTCGAAGAGGCCGTCAAGTGGATCTCTTACACCTACCTGTACGTCCGT
ATGAGGGCTAACCCTCTGGCCTACGGAATCTCTCACAAGGCCTACCAGATCGACCCCACCTTGAGGAAGCA
CCGCGAACAGCTGGTCATCGAAGTCGGACGCAAGCTGGACAAGGCCCAGATGATCCGCTTCGAAGAAAGG
ACCGGATACTTCTCCTCCACCGACCTCGGACGTACCGCCTCTCACTACTACATCAAGTACAACACTATCGAAA
CCTTCAACGAGTTGTTCGACGCCCACAAGACCGAAGGCGACATTTTCGCCATCGTGTCTAAGGCCGAAGAG
TTCGACCAGATCAAAGTCCGTGAAGAGGAAATCGAGGAACTGGATACCCTGCTGTCTAACTTCTGCGAACTG
TCTACCCCTGGCGGAGTCGAAAACTCTTACGGAAAGATCAACATCCTGCTCCAGACCTACATCTCCCGCGGA
GAAATGGACTCTTTCTCTCTGATCTCTGACTCCGCCTACGTGGCCCAGAACGCTGCTAGAATCGTCAGGGCT
CTGTTCGAGATCGCCCTGAGAAAGCGTTGGCCCACCATGACCTACCGCCTGCTGAACCTGTCTAAAGTCATC
GACAAGAGGCTGTGGGGATGGGCCTCTCCACTGAGGCAGTTCTCTATCCTGCCTCCTCACATCCTGACCAG
GCTGGAAGAGAAGAAGCTGACCGTTGACAAGCTGAAAGACATGCGCAAGGACGAGATCGGTCACATCCTCC
ACCACGTCAACATCGGCCTGAAAGTCAAGCAGTGTGTCCATCAGATCCCCAGCGTCATGATGGAAGCCTCTA
TCCAGCCTATCACCAGAACCGTCCTGAGGGTCACCCTGTCTATCTACGCCGACTTCACCTGGAACGACCAG
GTGCACGGAACCGTTGGAGAACCCTGGTGGATCTGGGTCGAAGATCCCACCAACGATCACATCTACCACTC
CGAATACTTCCTCGCGCTGAAGAAGCAAGTTATCTCCAAAGAAGCCCAGCTGCTGGTTTTCACTATCCCTATC
TTCGAACCCCTGCCTAGCCAGTACTACATCCGCGCTGTTTCTGACCGCTGGCTGGGAGCTGAAGCCGTCTG
CATCATCAACTTCCAACACCTCATCCTGCCAGAAAGGCACCCTCCACACACCGAGCTGTTGGACTTGCAGCC
TCTGCCAATCACCGCTCTCGGATGCAAGGCTTACGAGGCCCTGTACAACTTCAGCCACTTCAACCCCGTCCA
GACTCAGATCTTCCACACCTTGTACCACACCGACTGCAACGTCCTGCTGGGTGCTCCTACCGGATCTGGAAA
GACCGTCGCTGCCGAACTGGCTATCTTCCGCGTGTTCAACAAGTACCCCACCTCTAAGGCTGTCTACATTGC
CCCACTGAAGGCTCTCGTCAGGGAACGCATGGACGACTGGAAAGTCCGCATCGAGGAAAAGCTGGGCAAG
AAAGTGATCGAGCTGACCGGCGACGTCACCCCTGACATGAAGTCTATCGCCAAGGCCGACCTCATCGTCAC
TACCCCAGAAAAATGGGACGGTGTCTCTCGCTCTTGGCAGAACCGTAACTACGTCCAGCAAGTGACCATCCT
GATCATCGACGAAATCCACTTGCTCGGAGAAGAAAGGGGACCTGTCCTCGAAGTCATCGTCAGCCGCACCA

ACTTCATCTCTAGCCACACCGAAAAGCCCGTCCGTATCGTCGGACTCTCTACCGCCTTAGCTAACGCCAGAG
ACTTGGCCGACTGGCTGAACATCAAGCAAATGGGCCTGTTCAACTTCCGTCCATCTGTCAGGCCCGTGCCA
CTGGAAGTCCACATCCAAGGATTCCCCGGACAGCACTACTGCCCCAGGATGGCCTCAATGAACAAGCCTGC
CTTCCAGGCCATCCGCTCTCACTCTCCAGCTAAGCCTGTCCTGATCTTCGTCAGCTCTCGCAGGCAGACCA
GGCTCACCGCCTTGGAATTGATCGCTTTCCTGGCCACCGAAGAGGACCCCAAGCAGTGGCTCAACATGGAC
GAACGCGAGATGGAAAACATCATTGCCACCGTCCGCGACTCTAACCTCAAGCTGACCCTGGCTTTCGGAATC
GGAATGCACCACGCTGGACTGCACGAGAGGGACAGAAAGACTGTCGAAGAACTTTTCGTGAACTGCAAGGT
CCAGGTGCTGATCGCCACCTCTACTCTGGCTTGGGGTGTCAACTTCCCCGCTCACCTGGTTATCATCAAAGG
CACCGAGTACTACGACGGAAAGACCAGGCGCTACGTTGACTTCCCCATCACCGACGTCCTCCAGATGATGG
GTAGAGCTGGACGTCCTCAGTTCGACGACCAAGGCAAGGCTGTCATCCTGGTCCACGACATCAAGAAAGAC
TTCTACAAGAAATTCCTCTACGAGCCCTTCCCAGTCGAATCTTCCCTGCTGGGAGTCCTGAGCGACCACCTC
AACGCTGAAATTGCCGGCGGAACCATCACTTCTAAGCAGGACGCCCTCGACTACATCACCTGGACTTACTTC
TTCCGCCGCCTGATCATGAACCCCTCTTACTACAACCTGGGCGACGTGTCCCACGACTCTGTCAACAAGTTC
CTGTCTCACCTCATCGAAAAGTCCCTCATCGAGCTGGAACTGTCCTACTGCATCGAGATCGGAGAGGACAAC
CGCTCTATCGAGCCCCTGACCTACGGAAGGATCGCCTCCTACTACTACCTGAAGCACCAGACCGTCAAGATG
TTCAAGGACCGCCTGAAGCCTGAATGCTCTACCGAAGAACTGCTGTCCATCCTGTCCGACGCTGAAGAGTA
CACCGACCTGCCAGTCAGGCACAACGAGGATCACATGAACTCTGAACTGGCCAAGTGCCTGCCTATCGAGT
CTAACCCTCACTCCTTCGACTCTCCCCACACCAAGGCTCACCTCTTGCTGCAGGCTCACTTGTCTAGGGCCA
TGCTGCCTTGTCCTGACTACGACACCGACACCAAGACCGTGCTGGACCAAGCTCTCAGAGTCTGCCAGGCT
ATGTTGGACGTGGCCGCCAATCAAGGATGGCTCGTCACCGTCCTCAACATCACCAACCTGATCCAGATGGTC
ATCCAAGGCCGCTGGTTGAAGGACTCCTCTCTGCTCACCCTGCCTAACATCGAAAACCACCACCTCCACCTC
TTCAAGAAATGGAAGCCCATCATGAAGGGACCTCACGCCAGAGGAAGGACCTCCATCGAGTCTCTGCCCGA
ACTGATCCACGCCTGCGGAGGAAAGGACCACGTTTTCTCTTCTATGGTCGAATCCGAACTCCACGCCGCTAA
GACTAAGCAGGCTTGGAACTTCCTCTCTCACTTGCCTGTCATCAACGTCGGCATCTCCGTCAAAGGATCTTG
GGACGACTTGGTCGAAGGACATAACGAGCTGTCTGTCAGCACCCTGACCGCCGACAAAAGGGACGACAAC
AAATGGATCAAGCTCCACGCTGACCAAGAGTACGTCTTGCAAGTCTCTCTGCAGCGCGTCCACTTCGGATTC
CACAAGGGAAAGCCCGAATCTTGCGCTGTCACCCCTAGGTTCCCCAAGTCAAAGGACGAAGGCTGGTTCCT
GATCCTCGGAGAGGTGGACAAGCGCGAGCTGATCGCTCTTAAGCGCGTCGGTTACATCCGCAACCACCACG
TTGCCAGCCTGTCTTTCTACACCCCTGAGATCCCCGGACGCTACATCTACACCCTCTACTTCATGAGCGACT
GCTACCTGGGACTCGACCAGCAGTACGACATCTACCTGAACGTCACCCAGGCTTCTCTGTCTGCTCAAGTCA
ATACCAAGGTGTCCGACTCTCTGACCGACCTGGCTCTGAAGTAA

Synthetic gene sequence for expression of ASCC3 fragments

ATGGCACTTCCAAGGCTTACGGGTGCTTTGAGAAGTTTCAGCAACGTAACGAAGCAGGATAACTACAACGAG
GAAGTGGCAGACTTGAAGATCAAGCGAAGCAAGCTGCACGAACAAGTACTTGACCTTGGACTGACATGGAA
GAAGATCATCAAGTTCCTCAACGAGAAGCTCGAAAAGTCCAAGATGCAGTCCATCAACGAGGACCTCAAGGA
CATCCTCCACGCAGCAAAACAGATCGTGGGTACAGATAACGGTAGAGAGGCTATTGAGTCAGGTGCAGCATT
CCTTTTCATGACCTTCCACCTGAAGGACAGTGTAGGTCACAAGGAAACAAAGGCAATCAAGCAGATGTTCGG
TCCTTTCCCTAGTTCCAGCGCAACAGCAGCTTGTAACGCAACAAACAGAATCATCTCGCACTTCAGTCAGGA
CGACCTGACAGCACTTGTACAAATGACAGAGAAGGAGCACGGTGACAGAGTTTTCTTCGGTAAAAACCTCG
CTTTCAGTTTCGACATGCACGACCTTGACCACTTCGATGAGTTGCCAATCAACGGAGAAACACAGAAAACCA
TCAGCCTCGACTACAAGAAGTTCCTCAATGAGCACCTGCAAGAGGCATGTACACCAGAGTTGAAGCCAGTG
GAGAAAACTAACGGATCATTCCTGTGGTGTGAGGTGGAAAAGTACCTCAACAGTACCCTGAAGGAGATGACT
GAGGTGCCAAGGGTTGAAGATCTTTGTTGTACCCTTTACGACATGCTCGCTAGTATCAAGTCGGGTGACGAA
CTTCAGGACGAACTGTTCGAACTGCTGGGTCCAGAAGGACTTGAATTGATCGAAAAGTTGCTCCAGAACAG
GATAACCATCGTGGACAGATTCCTTAACTCCAGCAACGACCACAGGTTCCAAGCTCTTCAGGATAACTGCAA
GAAGATCCTGGGTGAAAACGCTAAGCCTAACTACGGATGCCAAGTGACTATCCAGTCAGAGCAAGAGAAGCA
GCTTATGAAGCAGTACAGACGTGAGGAGAAGAGGATCGCTAGGAGGGAAAAGAAAGCTGGTGAAGATCTGG
AAGTAAGTGAGGGTTTGATGTGCTTCGACCCAAAGGAACTGAGAATCCAGAGAGAACAGGCTCTGCTTAAC
GCTAGATCAGTGCCAATCCTGTCAAGACAAAGGGATGCTGATGTAGAGAAGATCCACTACCCTCACGTTTAC
GACTCACAGGCTGAAGCTATGAAAACATCGGCTTTCATCGCTGGTGCTAAGATGATCCTGCCTGAAGGAATC
CAAAGAGAAAACAACAAGCTCTACGAGGAAGTCCGTATCCCTTACTCGGAACCTATGCCACTGTCTTTCGAA
GAGAAGCCTGTGTACATCCAGGACTTGGACGAAATCGGACAACTTGCTTTCAAGGGTATGAAGAGGCTGAAC
CGTATCCAGTCAATCGTGTTCGAAACGGCTTACAATACCAACGAGAATATGCTGATCTGCGCCCCTACGGGA
GCTGGAAAAACTAACATCGCTATGCTGACTGTACTGCACGAAATCAGACAACATTTCCAGCAGGGAGTAATCA
AGAAGAACGAGTTCAAGATCGTGTACGTGGCCCCTATGAAAGCCCTGGCTGCTGAAATGACGGATTACTTCA
GCAGGAGACTGGAACCTTTGGGAATCATCGTAAAGGAACTGACAGGAGACATGCAGTTGAGCAAGTCAGAA
ATCCTGAGGACTCAGATGCTCGTGACTACTCCGGAAAAATGGGACGTTGTAACTCGTAAGTCAGTGGGAGAT
GTAGCTCTGTCTCAGATTGTCAGGCTGCTGATTCTCGATGAAGTACACCTCTTGCACGAAGATCGAGGTCCG
GTTTTAGAGTCTATTGTCGCTCGTACTCTCCGTCAAGTTGAATCTACTCAGTCTATGATTCGCATACTCGGACT
CTCTGCGACTCTTCCCAATTACCTTGACGTTGCCACTTTCTTGCACGTTAATCCGTACATTGGCCTGTTCTTTT
TCGATGGCAGATTCCGTCCCGTTCCCTTAGGACAAACCTTCCTCGGAATTAAGTGCGCCAATAAGATGCAGC
AGTTGAACAACATGGACGAGGTTTGCTACGAAAACGTCCTCAAACAGGTCAAAGCCGGTCATCAGGTTATGG
TCTTTGTCCACGCGCGAAATGCCACTGTTCGAACCGCGATGTCCTTAATTGAACGTGCCAAAAACTGTGGTC
ATATACCGTTCTTTTTCCCCACTCAAGGTCATGACTACGTTCTCGCCGAAAAACAGGTCCAACGATCTCGAAA
TAAGCAGGTCCGTGAACTCTTTCCCGATGGTTTTTCTATTCACCACGCCGGTATGTTGCGTCAAGATCGTAAC
TTGGTCGAAAACCTCTTCTCTAACGGCCATATTAAGGTCCTCGTCTGCACTGCGACTTTAGCCTGGGGCGTC

AATTTGCCCGCCCATGCCGTCATTATCAAAGGCACTCAAATATATGCCGCCAAACGTGGCTCTTTTGTGGATT
TGGGCATATTAGATGTGATGCAAATTTTTGGCCGCGCGGGCCGCCCGCAATTTGACAAATTTGGCGAAGGCA
TTATTATAACTACCCATGATAAACTGTCTCATTACCTCACCCTCCTCACCCAACGCAATCCCATTGAATCCCAAT
TTTTGGAATCCTTGGCGGATAACTTGAATGCCGAAATTGCCCTCGGCACCGTCACCAATGTCGAAGAAGCCG
TGAAATGGATTTCCTATACCTACTTGTACGTGCGAATGCGTGCGAATCCCTTAGCCTATGGCATTTCCCATAAA
GCGTATCAAATAGACCCCACCTTACGCAAACACCGCGAACAATTAGTCATTGAGGTCGGACGCAAATTGGATA
AAGCGCAAATGATACGCTTCGAGGAACGCACCGGCTATTTTTCCAGCACCGATTTGGGCCGCACCGCCAGC
CATTATTACATAAAATATAATACGATTGAAACCTTCAACGAGTTATTCGACGCCCATAAAACCGAGGGCGACATT
TTCGCCATTGTCAGCAAAGCCGAGGAGTTTGACCAAATCAAAGTCCGCGAGGAAGAGATTGAGGAGTTAGA
CACGTTATTATCGAATTTTTGCGAGTTATCGACGCCCGGCGGCGTGGAAAACAGCTACGGAAAGATCAACAT
CCTGCTCCAGACCTACATCAGCAGGGGTGAAATGGACAGTTTCTCGCTGATCAGTGACAGCGCTTACGTTGC
ACAAAACGCAGCTAGAATCGTAAGGGCTCTTTTCGAGATCGCACTTAGAAAGCGTTGGCCAACAATGACATA
CAGGCTGCTTAACCTGAGCAAGGTCATTGACAAGCGACTCTGGGGTTGGGCAAGTCCATTAAGGCAATTCA
GCATCCTTCCACCACATATCCTTACGAGACTGGAGGAGAAGAAGTTGACAGTGGACAAGTTGAAGGACATGC
GTAAGGACGAGATCGGTCACATCCTTCACCACGTTAATATCGGTCTGAAGGTCAAGCAGTGCGTGCATCAGA
TCCCAAGTGTTATGATGGAAGCCAGTATCCAGCCAATTACCCGTACGGTTCTGAGAGTAACACTCAGCATCTA
CGCAGACTTCACGTGGAACGATCAGGTTCACGGTACTGTTGGTGAACCTTGGTGGATTTGGGTAGAAGATCC
AACAAACGACCACATCTACCACAGTGAGTACTTCCTCGCACTTAAGAAGCAGGTCATCAGTAAGGAGGCACA
GCTTCTTGTCTTTACCATCCCCATCTTCGAGCCTCTTCCTAGCCAATACTACATCCGTGCAGTAAGCGACAGG
TGGTTAGGTGCAGAAGCAGTATGTATCATCAACTTCCAGCACCTTATCCTCCCAGAGAGGCATCCACCACATA
CAGAACTTCTGGACCTTCAGCCTTTGCCTATCACTGCACTGGGATGTAAAGCCTACGAGGCATTGTACAACTT
CAGTCACTTCAACCCAGTCCAGACGCAAATCTTCCACACGCTTTACCACACAGACTGTAACGTCCTTCTCGG
TGCTCCAACAGGTAGTGGAAAAACAGTAGCTGCTGAGTTGGCTATCTTCAGGGTTTTCAACAAGTACCCGAC
AAGCAAGGCTGTATATATCGCGCCTCTGAAGGCTCTGGTACGAGAAAGAATGGACGACTGGAAGGTACGAAT
CGAGGAAAAGCTGGGTAAAAAGGTCATCGAACTCACGGGTGACGTAACACCAGACATGAAGAGTATCGCTAA
GGCTGACCTTATCGTGACAACGCCTGAGAAGTGGGATGGAGTATCTAGGTCATGGCAAAACAGGAACTACGT
TCAGCAGGTAACGATCCTGATCATTGACGAGATCCACCTGTTGGGAGAGGAAAGAGGACCAGTTCTTGAAGT
GATCGTATCGAGAACCAACTTCATCAGCAGCCACACCGAGAAGCCAGTTAGGATCGTAGGTTTGTCAACCGC
TCTTGCTAACGCTAGAGACTTGGCTGATTGGCTTAACATCAAGCAGATGGGACTGTTCAACTTCCGTCCTAGC
GTTAGACCTGTACCTCTTGAGGTTCACATCCAGGGTTTCCCTGGTCAACATTACTGTCCTAGAATGGCTTCGA
TGAACAAGCCTGCTTTCCAGGCTATCAGATCGCATTCGCCTGCTAAACCTGTTCTTATCTTTGTCTCCTCCAG
GAGACAGACCAGACTGACTGCTCTGGAACTGATTGCCTTTCTGGCTACTGAAGAGGACCCTAAACAATGGTT
GAACATGGACGAGCGTGAAATGGAGAACATCATCGCTACCGTCCGAGATTCAAACTTGAAACTGACCCTGGC
TTTCGGAATCGGTATGCACCACGCTGGATTACACGAACGTGATCGAAAGACTGTTGAGGAATTGTTCGTGAA
CTGCAAGGTCCAGGTTTTGATTGCCACCTCGACTCTGGCCTGGGGAGTTAATTTCCCTGCCCATTTGGTCAT
CATAAAGGGAACTGAGTACTACGACGGAAAGACTCGTAGGTACGTTGACTTCCCCATTACTGACGTCTTGCA
GATGATGGGACGAGCCGGTCGTCCCCAATTTGATGATCAAGGAAAAGCCGTCATTTTGGTCCATGATATTAAG
AAGGACTTCTACAAGAAGTTCCTGTACGAGCCCTTCCCCGTGGAGTCATCGTTGTTGGGAGTGTTATCGGAC
CACCTGAACGCTGAAATTGCGGGTGGCACTATAACTTCTAAGCAGGATGCCTTGGATTACATTACCTGGACTT
ACTTCTTCCGCCGATTGATTATGAACCCCTCCTACTACAACCTCGGCGACGTGTCTCATGACTCAGTGAACAA
GTTCTTGTCCCACCTTATTGAGAAGTCCCTCATAGAACTCGAACTCTCCTACTGCATTGAGATTGGCGAGGAC
AACCGCTCCATAGAGCCCCTGACTTACGGTCGTATTGCGTCTTACTATTATCTGAAGCACCAGACTGTGAAGA
TGTTCAAGGACCGCTTGAAGCCTGAGTGTTCCACTGAGGAACTGTTGAGCATTCTGTCCGATGCCGAAGAAT
ACACTGACTTGCCGGTCAGACATAACGAAGATCACATGAACTCCGAACTGGCCAAGTGTCTGCCGATAGAAT
CAAATCCCCACTCTTTCGACTCCCCCCATACTAAAGCCCACTTACTCCTCCAAGCCCATTTGTCTCGTGCGAT
GTTACCCTGCCCCGATTATGATACTGATACTAAAACTGTCCTCGATCAGGCGTTACGTGTGTGCCAAGCCATG
TTAGATGTGGCGGCGAATCAAGGTTGGTTAGTGACTGTGCTGAATATTACCAATTTGATTCAGATGGTCATACA
GGGCCGTTGGTTGAAGGACAGCTCCTTACTCACCTTGCCGAATATAGAAAATCATCATCTCCACCTGTTCAAG
AAATGGAAACCGATTATGAAAGGCCCGCACGCCCGTGGCCGCACCTCTATAGAATCCCTCCCCGAATTAATA
CACGCGTGCGGTGGCAAAGATCATGTGTTTTCCTCTATGGTGGAATCTGAACTCCACGCCGCCAAAACCAAA
CAGGCGTGGAATTTTCTCTCACACCTGCCCGTCATTAATGTGGGCATTTCTGTCAAAGGCTCTTGGGATGATC
TCGTGGAAGGCCATAATGAATTATCAGTCTCTACCCTCACCGCCGATAAACGCGATGACAACAAATGGATTAA
GCTCCACGCCGACCAAGAATACGTCTTACAAGTCTCACTCCAACGCGTCCACTTTGGCTTTCACAAAGGCAA
ACCCGAATCATGCGCCGTGACCCCGCGCTTTCCCAAATCTAAAGATGAAGGCTGGTTTCTCATTTTAGGCGA
AGTGGATAAACGCGAATTAATTGCCCTCAAACGCGTCGGCTACATTCGCAACCACCACGTGGCCTCTCTCTC
TTTCTATACCCCCGAAATTCCGGGCCGCTATATATATACCCTCTATTTTATGTCAGACTGCTACCTCGGCCTCGA
TCAACAATATGACATATATCTGAATGTGACCCAAGCGTCACTCTCTGCCCAAGTGAATACCAAAGTCAGTGACT
CACTCACCGACCTCGCCCTGAAATAA

Synthetic gene sequence for expression of ASCC2

ATGCCAGCTCTTCCACTGGATCAGTTGCAGATCACACACAAAGATCCTAAGACGGGAAAGCTGAGGACTAGC
CCAGCACTGCACCCTGAGCAAAAGGCTGATAGATACTTCGTCCTGTACAAGCCACCACCTAAGGACAACATC
CCAGCTCTGGTTGAGGAGTACCTGGAGAGAGCAACATTCGTAGCAAACGACCTGGACTGGTTGCTTGCACT
TCCACACGACAAGTTCTGGTGCCAAGTAATCTTCGACGAAACCCTGCAAAAGTGCCTGGACAGCTACCTGA
GATACGTGCCAAGAAAGTTCGACGAGGGTGTAGCAAGCGCACCAGAAGTTGTTGACATGCAAAAGCGACTG
CATAGGAGCGTGTTCCTTACGTTCCTGAGGATGAGTACGCATAAGGAGAGTAAGGACCACTTCATCAGCCCT
AGCGCATTCGGAGAAATCCTTTACAACAACTTCCTGTTCGACATCCCGAAGATCCTCGACCTGTGCGTCCTG

TTCGGAAAGGGTAACAGTCCACTTCTCCAAAAGATGATCGGTAACATCTTCACCCAGCAGCCCAGTTACTACA<br>GCGACCTGGACGAAACGCTGCCAACAATCCTCCAAGTGTTCAGTAACATCCTTCAGCACTGCGGACTTCAG<br>GGTGACGGAGCAAATACGACACCACAAAAGCTGGAAGAACGTGGAAGGCTTACACCTAGTGACATGCCTCT<br>GCTTGAGCTGAAGGACATCGTACTTTACCTGTGCGACACCTGTACCACTCTCTGGGCATTTCTCGATATATTC<br>CCCCTCGCTTGTCAAACCTTCCAAAAGCACGACTTCTGCTACCGACTTGCTAGTTTCTACGAGGCCGCTATC<br>CCTGAGATGGAGTCAGCTATCAAGAAAAGGCGACTCGAAGATTCGAAGCTCCTCGGAGACTTGTGGCAAAG<br>GTTGTCACACTCAAGGAAGAAGCTCATGGAGATCTTCCACATCATCCTCAATCAGATCTGCCTCCTCCCGATA<br>CTTGAGTCCTCCTGTGACAACATACAGGGTTTCATTGAGGAGTTCCTCCAGATTTTCAGCTCCCTCCTCCAG<br>GAGAAGCGCTTTCTCCGTGACTACGACGCTCTCTTCCCTGTGGCTGAAGATATTAGCCTCCTCCAACAAGCC<br>TCAAGCGTATTGGACGAAACCAGAACCGCTTACATTCTTCAGGCTGTCGAATCGGCTTGGGAAGGTGTAGAC<br>AGAAGAAAGGCTACCGACGCTAAAGACCCTTCAGTGATTGAGGAGCCTAACGGAGAACCTAACGGTGTAAC<br>CGTGACAGCCGAAGCTGTTTCACAAGCGTCATCTCACCCTGAAAACTCTGAAGAGGAAGAATGTATGGGTG<br>CTGCGGCGGCTGTTGGACCTGCTATGTGTGGTGTCGAATTGGATTCTTTGATTTCTCAAGTCAAGGACTTGT<br>TGCCTGACTTGGGTGAGGGCTTCATTTTGGCCTGTTTGGAGTACTACCACTACGACCCGGAACAAGTGATAA<br>ACAACATTTTGGAGGAGCGATTGGCCCCGACCTTGTCTCAGTTGGACAGAAACTTGGACCGTGAGATGAAG<br>CCCGACCCGACTCCCTTATTAACCTCTCGACACAACGTGTTTCAGAACGACGAGTTTGACGTGTTTTCCAGG<br>GACTCTGTCGATTTATCCCGTGTGCATAAGGGCAAGTCCACCAGAAAAGAGGAGAACACTCGCTCCTTATTAA<br>ACGATAAACGAGCTGTGGCCGCCCAACGTCAAAGGTACGAGCAGTACTCTGTGGTTGTCGAAGAAGTCCCC<br>TTACAGCCCGGCGAATCTTTACCCTATCATTCGGTTTATTATGAAGATGAATATGATGATACTTATGATGGCAAT<br>CAGGTCGGCGCCAATGATGCCGATTCCGATGATGAATTAATTTCCCGTCGTCCCTTTACTATTCCCCAGGTCC<br>TGCGTACTAAAGTCCCGCGCGAAGGCCAGGAGGAAGATGACGACGATGAAGAAGATGATGCCGATGAAGAA<br>GCGCCCAAACCGGATCACTTTGTCCAGGACCCCGCCGTTTTACGCGAAAAAGCCGAAGCCCGCAGGATGG<br>CGTTTTTAGCCAAAAAAGGCTATCGCCACGATTCGTCCACTGCCGTTGCCGGCTCACCCAGAGGTCATGGC<br>CAGTCTCGTGAAACAACTCAGGAAAGAAGAAAAAAAGAAGCGAATAAAGCGACTCGTGCCAATCACAATCGC<br>CGTACTATGGCCGATCGCAAACGCTCGAAAGGCATGATTCCCTCTTAA

[a] Expression of ASCC3^{FL} and ASCC3 fragments was based on different synthetic gene sequences.

Supplementary Table 4: Nucleic acids used in helicase assays[a].

**3’-overhang DNA**
(Atto 540 Q)5’-**GGCCGCGAGCCG**GAAATTTAATTATAAACCAGACCGTCTCCTC-3’
5’-**CGGCTCGCGGCC**-3’(Alexa Fluor 488)
**5’-overhang DNA**
5’-CTCCTCTGCCAGACCAAATATTAATTTAAAG**GCCGAGCGCCGG**-3’(Atto 540 Q)
(Alexa Fluor 488)5’-**CCGGCGCTCGGC**-3’
**3’-overhang RNA**
(Atto 540 Q)5’-**GGCCGCGAGCCG**GAAAUUUAAUUAUAAACCAGACCGUCUCCUC-3’
5’-**CGGCUCGCGGCC**-3’(Alexa Fluor 488)

[a] Duplex regions in bold.

Supplementary Figures

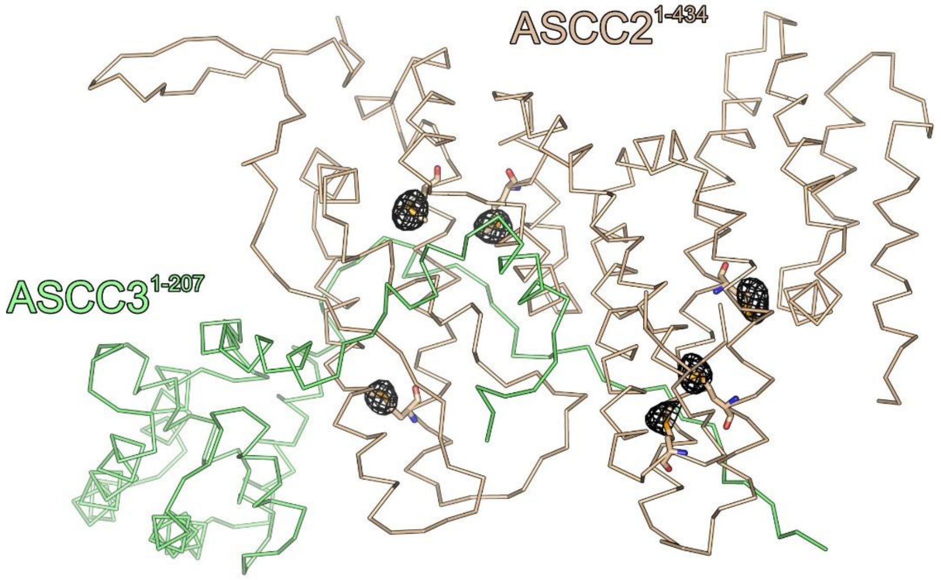

Supplementary Figure 1: Anomalous difference Fourier map.

Anomalous difference Fourier map contoured at the 5 σ level (gray mesh), showing the positions of selenium atoms in the side chains of SeMet residues (shown as sticks). View as in Fig. 1d,f, left. ASCC2$^{1-434}$, beige; ASCC3$^{1-207}$, lime green.

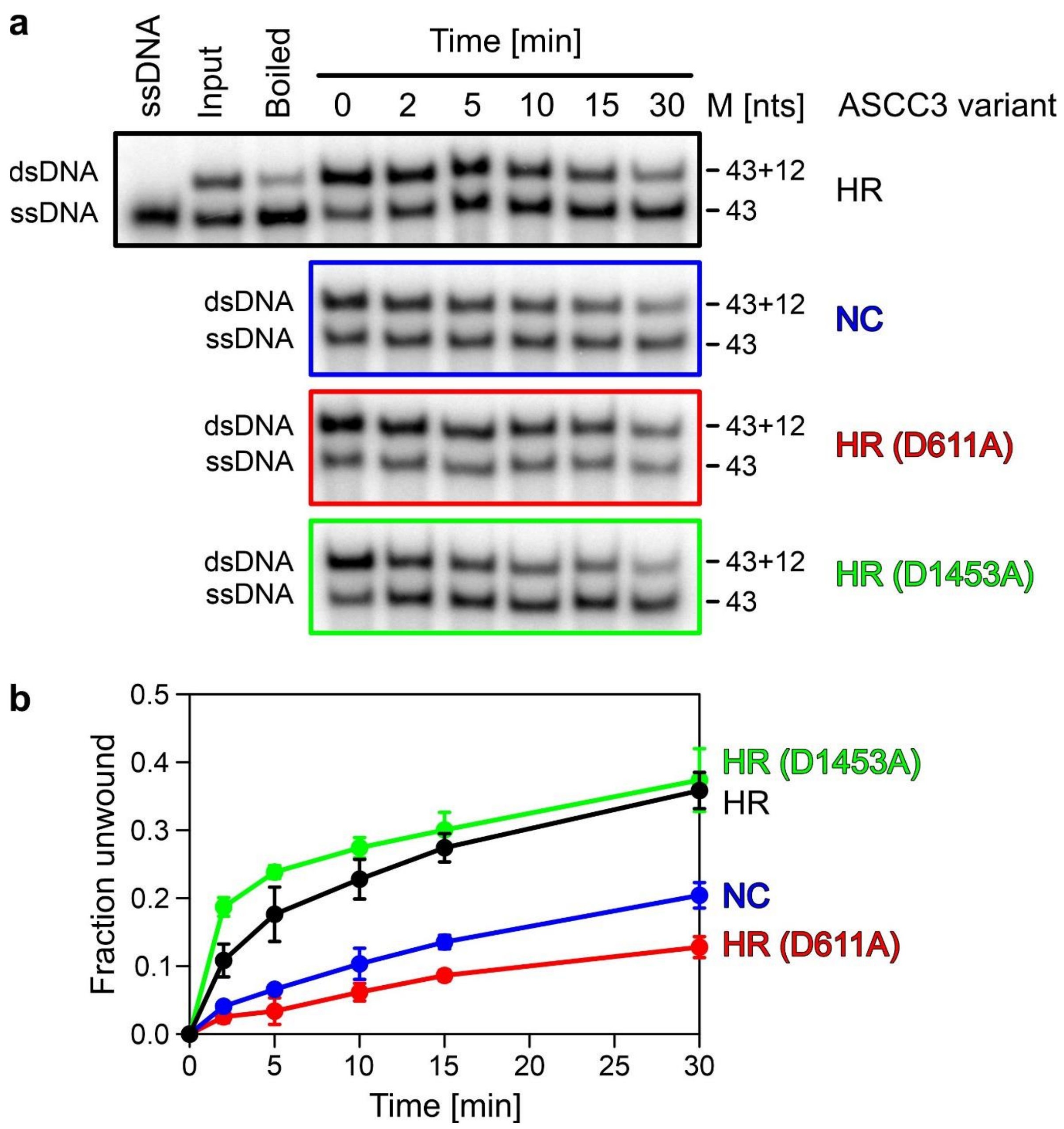

Supplementary Figure 2: Gel-based unwinding assay.

a, Gels showing time courses of unwinding of a 3'-overhang DNA substrate (long strand radiolabeled; same sequence as used in stopped-flow/fluorescence-based unwinding assays; Supplementary Table 4) by the ASCC3 variants indicated on the right. HR / NC / HR (D611A) / HR (D1453A), ASCC3 variants as defined in the text. Bands representing double-stranded (ds) and single-stranded (ss) DNA are labeled on the left. The DNA input contained a mixture

of dsDNA and ssDNA. Boiled, DNA sample heated to 95 °C for 5 minutes before gel analysis. M [nts], molecular weight marker (number of nucleotides). Lanes representing $ASCC3^{HR}$ and $ASCC3^{NC}$ were run on the same gel, and lanes representing $ASCC3^{HR,D611A}$ and $ASCC3^{HR,D1453A}$ were run on the same gel, and gels were processed in parallel. Each gel contained marker lanes “ssDNA”, “Input” and “Boiled”, identifying running positions of dsDNA and ssDNA, but are only shown for the first panel. Complete gels are shown in the source data provided for this paper. To facilitate comparison, relevant gel sections are displayed below each other. **b**, Quantification of the data shown in (**a**). Data represent means ± SD of n=3 independent experiments, using the same biochemical samples.

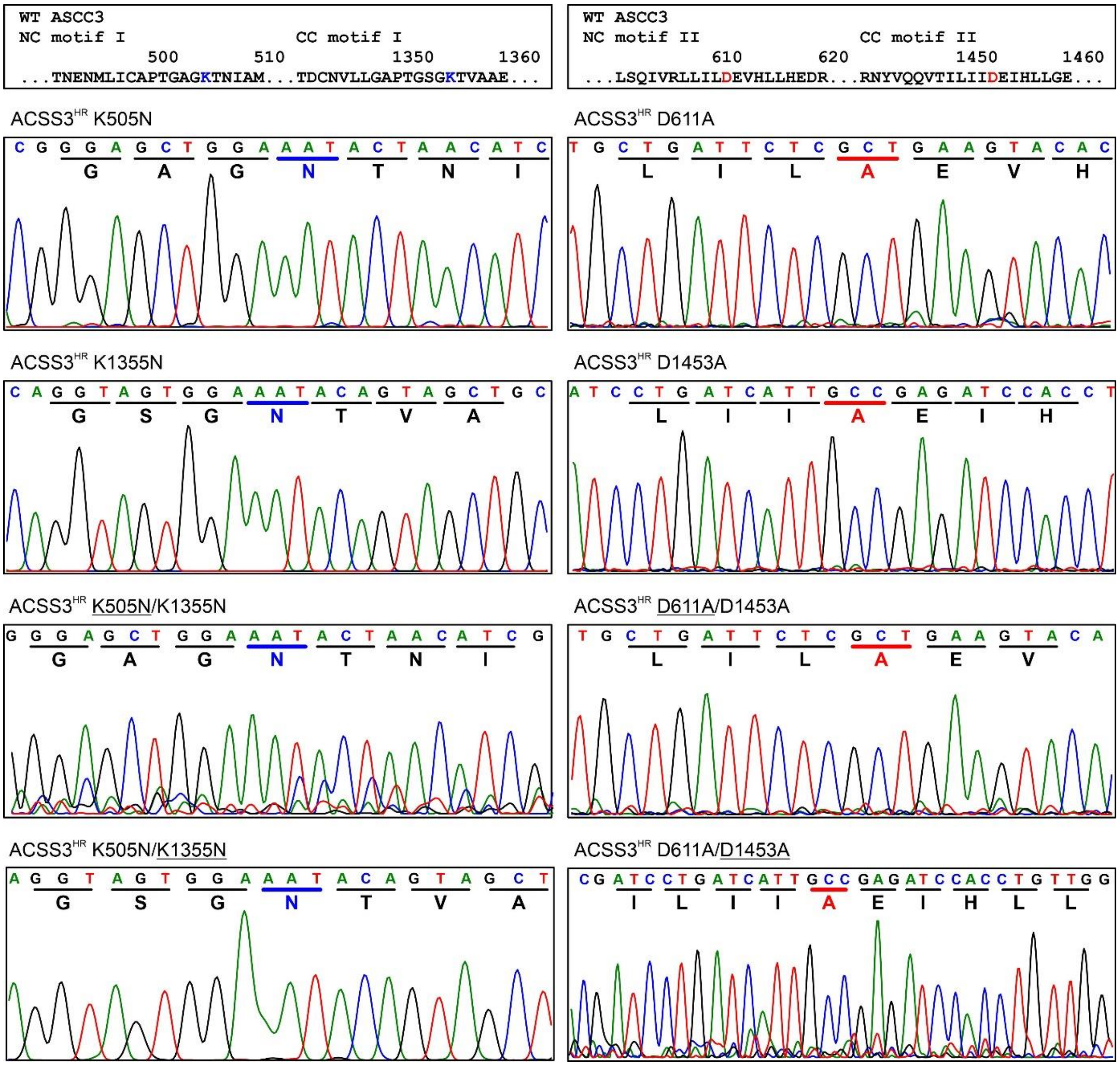

Supplementary Figure 3: Sequencing results.

Sequencing results documenting the presence of the intended mutations and the encoded residues in the baculoviruses expressing ASCC3^{HR} variants for helicase activity assays shown in Fig. 6b-d. Top panel, motif I (left) and motif II (right) sequences in WT ASCC3; conserved K and D residues highlighted blue and red, respectively. The ASCC3^{HR} variants encoded by the sequenced baculoviruses are indicated above the panels. For baculoviruses encoding ASCC3^{HR} variants with double residue substitutions, sequencing results for the underlined

substitution is displayed in the respective windows. NC, N-terminal helicase cassette; CC, C-terminal helicase cassette.
