# Supplementary material for: The interaction of DNA repair factors ASCC2 and ASCC3 is affected by somatic cancer mutations
Source: Nat Commun. 2020 Nov 2;11:5535. doi: 10.1038/s41467-020-19221-x (PMC7608686; doi:10.1038/s41467-020-19221-x)
Supplement: Supplementary file 3 — Reporting Summary [file 41467_2020_19221_MOESM3_ESM.pdf]

nature research

Corresponding author(s): Markus C. Wahl
Last updated by author(s): Sep 23, 2020

Reporting Summary

Nature Research wishes to improve the reproducibility of the work that we publish. This form provides structure for consistency and transparency in reporting. For further information on Nature Research policies, see our Editorial Policies and the Editorial Policy Checklist.

Statistics

For all statistical analyses, confirm that the following items are present in the figure legend, table legend, main text, or Methods section.

n/a	Confirmed	
[ ]	[x]	The exact sample size (n) for each experimental group/condition, given as a discrete number and unit of measurement
[ ]	[x]	A statement on whether measurements were taken from distinct samples or whether the same sample was measured repeatedly
[x]	[ ]	The statistical test(s) used AND whether they are one- or two-sided<br>*Only common tests should be described solely by name; describe more complex techniques in the Methods section.*
[x]	[ ]	A description of all covariates tested
[x]	[ ]	A description of any assumptions or corrections, such as tests of normality and adjustment for multiple comparisons
[ ]	[x]	A full description of the statistical parameters including central tendency (e.g. means) or other basic estimates (e.g. regression coefficient) AND variation (e.g. standard deviation) or associated estimates of uncertainty (e.g. confidence intervals)
[x]	[ ]	For null hypothesis testing, the test statistic (e.g. F, t, r) with confidence intervals, effect sizes, degrees of freedom and P value noted<br>*Give P values as exact values whenever suitable.*
[x]	[ ]	For Bayesian analysis, information on the choice of priors and Markov chain Monte Carlo settings
[x]	[ ]	For hierarchical and complex designs, identification of the appropriate level for tests and full reporting of outcomes
[x]	[ ]	Estimates of effect sizes (e.g. Cohen's d, Pearson's r), indicating how they were calculated

Our web collection on statistics for biologists contains articles on many of the points above.

Software and code

Policy information about availability of computer code

Data collection	XDS, version Jan 26, 2018, diffraction data procession software
Data analysis	Phenix, version 1.17.1, crystallographic software<br>Coot, version 0.9, model building software<br>PyMOL, version 1.8, molecular graphics software, Schrödinger, LLC<br>MicroCal, version MAN0579-03-EN-00, November 2018, PEAQ-ITC analysis software, Malvern Panalytical<br>ImageJ, version 1.52q, image analysis software<br>ImageQuant, version 5.2, image analysis software, Cytiva<br>Prism version 8, analysis and graphing software, GraphPad

For manuscripts utilizing custom algorithms or software that are central to the research but not yet described in published literature, software must be made available to editors and reviewers. We strongly encourage code deposition in a community repository (e.g. GitHub). See the Nature Research guidelines for submitting code & software for further information.

Data

Policy information about availability of data

All manuscripts must include a data availability statement. This statement should provide the following information, where applicable:
- Accession codes, unique identifiers, or web links for publicly available datasets
- A list of figures that have associated raw data
- A description of any restrictions on data availability

Structure factors and coordinates have been deposited in the RCSB Protein Data Bank (https://www.rcsb.org/) with accession code 6YXQ (https://doi.org/10.2210/

pdb6YXQ/pdb). Source data for Figs. 1a-c, 3a-c, 3i-j, 5c-h, 6 and Supplementary Figure 2 are provided. Other data are available from the corresponding author upon reasonable request.

Field-specific reporting

Please select the one below that is the best fit for your research. If you are not sure, read the appropriate sections before making your selection.

☒ Life sciences ☐ Behavioural & social sciences ☐ Ecological, evolutionary & environmental sciences

For a reference copy of the document with all sections, see nature.com/documents/nr-reporting-summary-flat.pdf

Life sciences study design

All studies must disclose on these points even when the disclosure is negative.

Sample size	For crystallographic analyses, near-complete and redundant diffraction data sets were obtained from single crystals, which is customary procedure.<br>For interaction tests, sample sizes were designed to provide significant and reproducible signals (ITC; analytical SEC) or to provide clearly visible and quantifiable bands on gels (SDS PAGE analysis of analytical SEC; WB analysis of pull-down experiments; gel-based unwinding assays).<br>For stopped-flow/fluorescence-based unwinding assays, sample sizes were adjusted to provide significant and reproducible fluorescence signals.
Data exclusions	No data were excluded from the analyses.
Replication	For interaction tests and unwinding assays at least two independent experiments were performed using the same biochemical samples (such as recombinant proteins, nucleic acids, cell lines, antibodies). All attempts at replication were successful.
Randomization	This study reports results from rationally designed in vitro biochemical/biophysical experiments, for which randomization is not applicable, as there is no danger of confounding independent variables in the experimental design.
Blinding	This study reports results from rationally designed in vitro biochemical/biophysical experiments, for which blinding is not applicable, as the experiments did not involve human subjects, and as the results from the experiments can be objectively evaluated/quantified.

Reporting for specific materials, systems and methods

We require information from authors about some types of materials, experimental systems and methods used in many studies. Here, indicate whether each material, system or method listed is relevant to your study. If you are not sure if a list item applies to your research, read the appropriate section before selecting a response.

Materials & experimental systems

n/a	Involved in the study	
☐	☒	Antibodies
☐	☒	Eukaryotic cell lines
☒	☐	Palaeontology and archaeology
☒	☐	Animals and other organisms
☒	☐	Human research participants
☒	☐	Clinical data
☒	☐	Dual use research of concern

Methods

n/a	Involved in the study	
☒	☐	ChIP-seq
☒	☐	Flow cytometry
☒	☐	MRI-based neuroimaging

Antibodies

Antibodies used	Antibodies against ASCC2 (Proteintech, 11529-1-AP) and the Flag tag (Sigma-Aldrich, F3165)
Validation	Proteintech, 11529-1-AP:<br>https://www.ptglab.com/Products/ASCC2-Antibody-11529-1-AP.htm#validation<br>Among other validations: Various lysates were subjected to SDS PAGE followed by western blot with 11529-1-AP (ASCC2 antibody) at dilution of 1:1000 incubated at 4°C overnight<br><br>Sigma-Aldrich, F3165:<br>https://www.sigmaaldrich.com/catalog/product/sigma/f3165?lang=de&region=DE&gclid=EAIaIQobChMIhcnTs-G-6QIVh-F3Ch2giwK7EAAYASAAEgJ6n_D_BwE

Eukaryotic cell lines

Policy information about cell lines

Cell line source(s)	HEK293 Flp-In T-REx cells (Invitrogen)<br>Sf9 cells (Thermo Fisher Scientific)<br>High Five cells (Thermo Fisher Scientific)
Authentication	Parental HEK293 Flp-In T-REx cells were purchased from the company and were not further authenticated. Expression of transgenes was confirmed by western blot.<br>Parental Sf9 cells and High Five cells were purchased from the company and were not further authenticated. Baculovirus-based production of target proteins was confirmed by detecting target proteins on SDS PAGE gels after affinity purification.
Mycoplasma contamination	HEK293 Flp-In T-REx cell lines were tested for mycoplasma contamination and confirmed to be negative.<br>Sf9 cells and High Five cells were not tested for for mycoplasma contamination, as cells were only used for recombinant protein production.
Commonly misidentified lines<br>(See ICLAC register)	No commonly misidentified cell lines were used in the study.
